# High aerospora levels and associated atmospheric circulation patterns: Pretoria, South Africa

**DOI:** 10.1007/s00484-024-02781-8

**Published:** 2024-09-28

**Authors:** S. J. Roffe, L. B. Ajikah, J. John, R. M. Garland, K. Lehtipalo, M. K. Bamford

**Affiliations:** 1 Agrometeorology Division, Agricultural Research Council-Natural Resources and Engineering, Pretoria, South Africa; 2 Department of Geography, University of the Free State, Bloemfontein, South Africa; 3 Global Change Institute, University of the Witwatersrand, Johannesburg, South Africa; 4 Evolutionary Studies Institute, University of the Witwatersrand, Johannesburg, South Africa; 5 Department of Botany, University of Calabar, Calabar, Nigeria; 6 Institute of Atmospheric and Earth System Research, University of Helsinki, Helsinki, Finland; 7 SMART Places, Council for Scientific and Industrial Research, Pretoria, South Africa; 8 Department of Geography, Geoinformatics and Meteorology, University of Pretoria, Pretoria, South Africa

**Keywords:** Airborne pollen and spores (aerospora), Allergenicity, High-risk days, Atmospheric circulation classification, Pretoria

## Abstract

At high exposure levels, airborne pollen grains and fungal spores (termed aerospora hereafter), can trigger severe allergic respiratory diseases. For South Africa’s administrative capital Pretoria, which boasts dense vegetation within a large urban forest, it is valuable from a health perspective to understand daily atmospheric circulation patterns associated with high aerospora levels. Therefore, we utilised a daily aerospora grain count dataset collected in Pretoria from 08/2019–02/2023 to investigate atmospheric circulation patterns (derived from ERA5 reanalysis sea level pressure [SLP] and 500 hPa geopotential height [zg500] fields) associated with high-risk aerospora levels (aerospora grain count > 90th percentile). Concentrated during October-May, there were 128 high-risk days, with 69.6% of days occurring in November, February and April. Although generally above-average mid-tropospheric subsidence levels prevailed over Pretoria during high-risk days, no single distinct atmospheric circulation pattern was associated with these high-risk days. Therefore, using Principal Component Analysis, we classified 14 Circulation Weather Types (CWTs) for October-May months between 08/2019–02/2023 to assess which CWTs most frequently occurred during high-risk days. Three CWTs had a statistically significant proportion of high-risk days – collectively they occurred during 37.1% of days studied, yet accounted for 45.3% of high-risk days. Among these CWTs, two CWTs were similarly associated with surface and mid-tropospheric high-pressure conditions, while the third was associated with a surface and mid-tropospheric trough. By comparing our CWT classification to daily synoptic charts (from the South African Weather Service), our classification can be used to identify days with potentially high allergenicity risk over Pretoria.

**Supplementary Information:**

The online version contains supplementary material available at 10.1007/s00484-024-02781-8.

## Introduction

Although various mechanisms disperse pollen grains and fungal spores, it is predominantly through wind dispersal that they become airborne (Esterhuizen et al. 2023; Uguz 2023). Globally, airborne pollen grains and fungal spores (termed aerospora hereafter) are known as significant bioaerosols, inducing allergies among susceptible individuals (Singh and Mathur 2012; Ajikah et al. 2021, 2023). Aerospora can trigger many types of respiratory conditions (e.g., allergic rhinitis, conjunctivitis, asthma, and atopic dermatitis), and at high levels, aerospora can be particularly dangerous for allergy sufferers (Asher and Weiland 1998; Steckling-Muschack et al. 2021). For allergy sufferers, the inhalation of an offending aeroallergen leads to the production of IgE antibodies, and with ongoing exposure, cross-linking of allergen-specific IgE molecules on mast cells occurs (Singh and Mathur 2012). When mast cells degranulate, they release histamine and other inflammatory mediators/chemicals which triggers common allergy symptoms, including wheezing, runny nose, itching, rashes, and sneezing (Singh and Mathur 2012). These symptoms range from mild to life-threatening and could be localised or systemic (Singh and Kumar 2004).

**Fig. 1:**
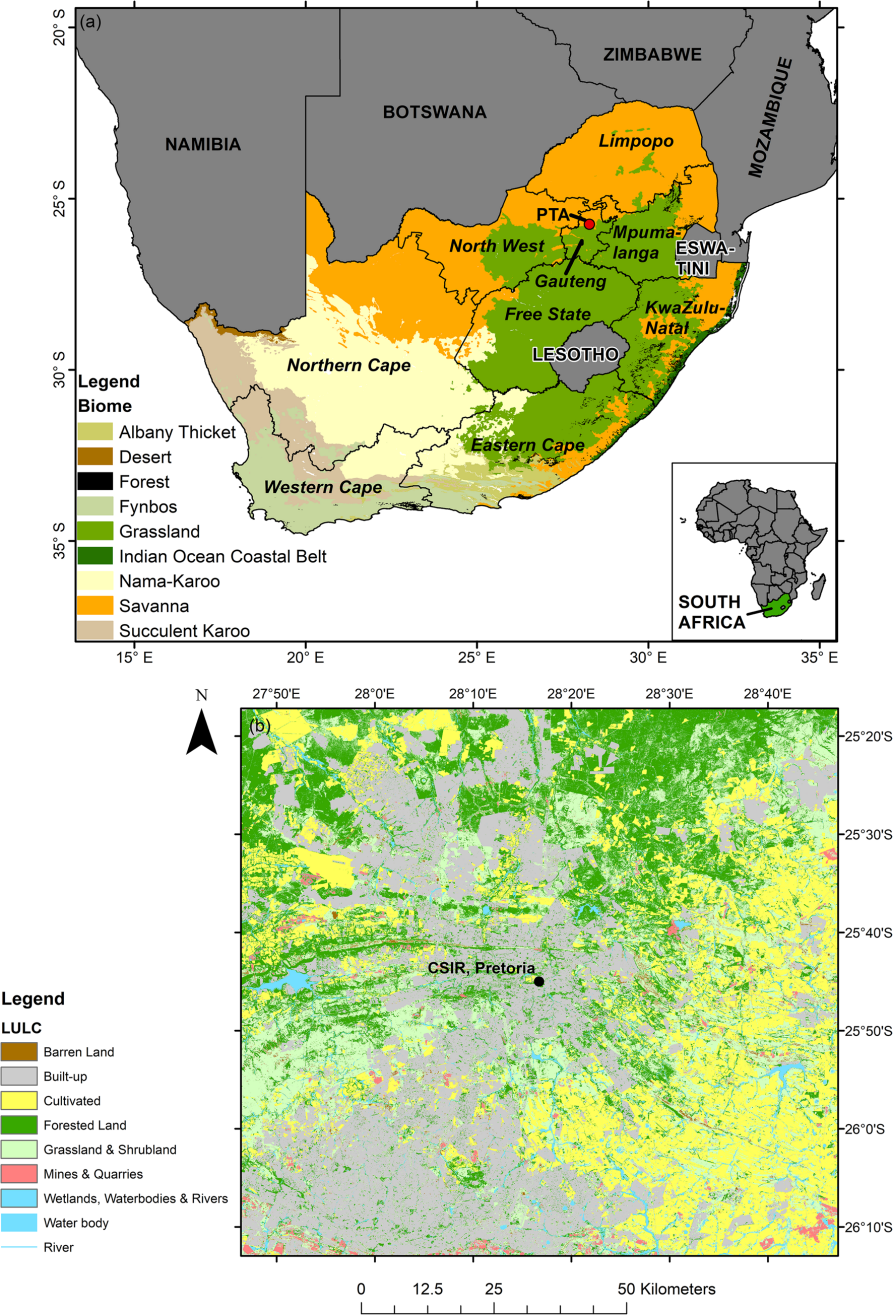
Map of the study area depicting (a) Pretoria (as PTA) within South Africa’s Gauteng province and Grassland and Savanna biome, and (b) the location of the Council for Scientific and Industrial Research (CSIR) 7-day volumetric spore trap (-25.75°S 28.28°E at 1396 m.asl) within Pretoria, showing the surrounding area. Biome data are sourced from the South African National Biodiversity Institute and represent the biome distribution for 2018 (SANBI 2018). Land use and land cover (LULC) data are sourced from the South African Department of Forestry, Fisheries and the Environment and represent that recorded from 1 January to 31 December 2020 (DFFE 2021)

Aeroallergen exposure impacts and affects developed and developing countries, and both are experiencing an increase in respiratory allergies, collectively escalating socio-economic costs to the healthcare system worldwide (Bousquet et al. 2020; Dierick et al. 2020). South Africa is among the countries experiencing an increase in the incidence and severity of asthma and allergic diseases such as allergic rhinitis (Green et al. 2013; Esterhuizen et al. 2023). Although there is some uncertainty in the statistics, within South Africa, allergic rhinitis is estimated to affect ~ 20–30% of the population, with co-morbid asthma evident in 20% of cases (Ajikah et al. 2020). Allergic rhinitis and asthma are most commonly triggered by pollen and house dust mite allergens, being common aeroallergens in South Africa (Ajikah et al. 2020; Potter 2010). Most common global aeroallergens occur within South Africa because the country hosts a variety of climate types, manifesting as a variety of biomes (Potter 2010; Fig. 1a). Furthermore, in many regions of South Africa, non-native trees (e.g., eucalypt, *Eucalyptus* spp. [L Hér.] and pine, *Pinus* spp. [L]) have been planted to produce timber and for other uses, such as for ornamental value, and they also have undesired pollen allergenicity effects (Ajikah et al. 2020). Within South Africa, exotic trees comprise a large proportion of the main pollen types recovered from aerospora sampling, however, compared to many northern hemisphere cities, lower levels are recorded (Esterhuizen et al. 2023). Within many regions of South Africa, grass pollen is commonly dispersed by wind and can trigger severe allergic diseases (Esterhuizen et al. 2023).

To understand health risks associated with aerospora, research has been undertaken to understand environmental factors influencing aerospora levels (Alarcón et al. 2023). Findings from many of these studies reflect that aerospora emission depends on environmental characteristics (e.g., vegetation cover and land use), plant flowering periods and plant responses to local atmospheric conditions, of rainfall, relative humidity, temperature, and winds, which are associated with large-scale atmospheric circulation patterns (Bishan et al. 2020; Grinn-Gofroń et al. 2020; Ajikah et al. 2023; Alarcón et al. 2023). For example, in the city neighbouring the study region of Pretoria (i.e., in Johannesburg), Ajikah et al. (2023) demonstrated that rainfall, relative humidity, and temperature were key factors influencing daily aerospora levels from 08/2019–07/2021. They showed that higher aerospora levels were generally associated with low/moderate rainfall (< 20 mm), relatively warm daily average temperatures (~ 15–20˚C), and mid-ranging relative humidity levels (~ 40–60%; Ajikah et al. 2023). These conditions are more generally associated with high-pressure systems prevailing over the Gauteng Province, where our study region is also located (Tyson and Preston-Whyte 2000). These high-pressure conditions generally promote calm and stable atmospheric conditions which can limit dispersal, and potentially promote accumulation, of aerospora, especially in areas with high vegetation density, such as our study region (Tyson and Preston-Whyte 2000; Fig. 1b). In terms of atmospheric circulation patterns, over Barcelona, Spain, Alarcón et al. (2023) similarly demonstrated that stagnant conditions associated with high-pressure systems generally link to higher aerospora levels. Such conditions are also frequently associated with higher air pollution levels over much of the South African Highveld region where Gauteng is located (e.g., Tyson and Preston-Whyte 2000; Jury 2017; Matandirotya and Burger 2021, 2023). Consequently, it can be hypothesised that such conditions are associated with higher aerospora levels, linking to higher health risks; however, to date, no study has assessed the link between atmospheric circulation and high aerospora levels to provide evidence towards this hypothesis.

In response to the many unknowns associated with aerospora patterns in South Africa and considering the associated health dangers after exposure (especially at elevated levels), the South African Pollen Network (SAPNET) was established in 08/2019 to conduct long-term national aerospora monitoring throughout South Africa (Berman et al. 2020; Ajikah et al. 2020). Internationally recognised monitoring is continuously carried out, using 7-day volumetric Burkard spore traps, in seven of South Africa’s cities, including Bloemfontein, Cape Town, Durban, Gqeberha (formerly Port Elizabeth), Johannesburg, Kimberley and Pretoria (Berman et al. 2020; Ajikah et al. 2020). Daily counts are then communicated in weekly reports, shared online on the SAPNET website (https://pollencount.co.za/), to provide an allergy risk guide for the public and healthcare providers within the respective cities. For healthcare providers, these reports are particularly useful for making clinical diagnoses and decisions. The SAPNET website also displays the flowering periods of plants and seasonal occurrences of aerospora to enable forecasting and predicting future occurrences. Therefore, the information on the SAPNET website is intended to serve as an early warning system to address the problems of allergic diseases caused by aerospora.

Since the inception of SAPNET, several studies have been conducted to provide insight into the types of aerospora that occur within monitoring locations (Berman et al. 2020), characterise aerospora levels, develop aerospora calendars, highlight how geographic differences are important determinants of aerospora types and concentrations (Esterhuizen et al. 2023), and demonstrate how variations in meteorological parameters (e.g., rainfall, temperature, relative humidity, and wind speed and direction) influence aerospora levels (Ajikah et al. 2023). Indeed, much work has been undertaken; however, the SAPNET monitoring network data still provide opportunities to study many more topics of importance. One such topic that has not been considered to date is the influence of atmospheric circulation patterns on aerospora levels, considering either individual types or the collective aerospora sample (e.g., Ojrzyńska et al. 2020; Paschalidou et al. 2020). Another is a focus on the source regions of aerospora through trajectory modelling (e.g., Makra et al. 2010). These are topics that have been evaluated in a similar context for air pollution in South Africa (e.g., Hersey et al. 2015; Matandirotya and Burger 2021, 2023; Jury and Buthelezi 2022), and since aerospora represent biological air pollutants, these are important studies to learn from. Thus, for this study, as highlighted above, we focus on Pretoria and consider these important research avenues that have not yet been explored using the SAPNET data. We focus on the Pretoria region because it represents a highly populated city within Gauteng, which is South Africa’s most populated province (GCRO 2018). Moreover, Pretoria is among the regions in South Africa where ambient air pollution levels for many pollutants are non-compliant with the National Ambient Air Quality Standards (e.g., Altieri and Keen 2019; DFFE 2023; Howlett-Downing et al. 2023). Furthermore, from an international point of view, a focus on Pretoria provides a comparative location for regions with similar climatic and environmental conditions. Therefore, our aim was to determine atmospheric circulation patterns associated with high aerospora levels in Pretoria, South Africa, from 08/2019–02/2023. We refer to these high levels as high-risk as these are levels that could potentially trigger allergies among individuals in Pretoria. Thus, by considering high-risk days, we aim to assist public health advisories and benefit allergy sufferers within Pretoria by identifying atmospheric circulation patterns that are more likely to trigger allergies. This can in turn inform public health policies not only locally for Pretoria and other similar regions in South Africa, but also for similar international regions.

## Materials and methods

### Study site

The City of Pretoria (also known as the City of Tshwane) spans an area of ~ 6368 km^2^ and is located at ~ 25.7°S and ~ 28.2°E (at 1339 m.asl) within South Africa’s Gauteng Province (Fig. 1). It forms part of the larger Gauteng City-Region, representing South Africa’s economic hub, and it hosts a population of ~ 4 million people (GCRO 2018).

The City has a warm, temperate climate (Cwb Köppen-Geiger climate type), with dry, cool winters (average temperature of ~ 12 °C and average rainfall of ~ 100 mm) and warm, wet summers (average temperature of ~ 20 °C and an average rainfall of ~ 550 mm; Engelbrecht and Engelbrecht 2016; Landman et al. 2017). The city has an annual average rainfall total of ~ 650 mm, while the annual average temperature is ~ 18 °C (Landman et al. 2017).

Pretoria is situated within South Africa’s Grassland and Savanna biomes (Fig. 1a; Mucina et al. 2006). More specifically, the surrounding vegetation on the Council for Scientific and Industrial Research (CSIR) campus, where the volumetric spore trap is located (Fig. 1b), includes a variety of mostly indigenous trees and grasses. The baseline vegetation for the site is Marikana Thornveld (SVCB; Mucina et al. 2006). Many of the common species typical of this vegetation are found on the CSIR campus. This includes tree species such as *Acacia burkei* (Benth.)*, Acacia Karroo* (Hayne)*, Combretum molle* (R. Br. ex G. Don.)*, Rhus lancea* (L.f.)*, Ziziphus mucronata* (Wild)*, Acacia Nilotica* (L.Wild. ex Delile)*, Celtis africana* (Burm. f.), and grass species such as *Elionurus muticus* (Spreng)*, Eragrostis lehmanniana* (Schrad)*, Setaria sphacelata* (Schumach)*, Themeda triandra* (Forssk)*, Aristida scabrivalvis subsp. scabrivalvis* (Hack)*, Fingerhuthia africana* (Nees ex Lehm)*, Heteropogon contortus* (L.)*, Hyperthelia dissoluta* (Nees ex Steud)*, Melinis nerviglumis* (Franch)*,* and *Pogonarthria squarrosa* (Stapf; Mucina et al. 2006). Horticultural and ornamental species dominate in the urbanised and residential areas of Pretoria.

### Aerospora data

To produce daily aerospora records for Pretoria, a 7-day Hirst-type volumetric pollen and fungal spore trap was set up on the CSIR campus (at -25.75°S 28.28°E at 1396 m.asl) in 08/2019 as part of the SAPNET aerospora monitoring initiative across South African cities (Fig. 1b; Ajikah et al. 2020; Berman et al. 2020); each of these samplers have a constant flow rate set at 10 L.min^−1^ and a drum rotation of 2 mm.hr^−1^ (Berman et al. 2020). This sampler is a standard aerospora monitoring device, manufactured by Burkard Manufacturing in the UK, and is widely used in aerobiological studies (Cadman et al. 1997; Berman 2007; Bishan et al. 2020). In addition to the standard sampler, the preparation, counting and aerospora identification process undertaken to produce daily samples is standard for all SAPNET monitoring sites (see Ajikah et al. 2023). From the spore trap, daily aerospora samples are collected using Melinex tape coated with Vaseline® jelly. The tape is then cut into seven segments and mounted on slides with coverslips using glycerol jelly media. Thereafter, aerospora grains are counted (with three longitudinal traverses read and counted per slide, avoiding the margins) using a ZEISS Axioscope 5 microscope at × 400 magnification (Esterhuizen et al. 2023), while their identification is based on their morphological characteristics and published works (e.g., Berman 2018; Scott 1982).

The resulting sample used herein represents the daily sum of aerospora grains for all recorded aerospora types, spanning the period 08/2019–02/2023; the daily count of aerospora grains, without conversion to an aerospora concentration, was used for analysis. Similar to the Johannesburg daily aerospora sample utilised by Ajikah et al. (2023), the resulting dataset has ~ 20% missing data which coincides with the South African Covid-19 pandemic lockdown periods, and no gap-filling process was undertaken.

### Atmospheric circulation data

To characterise the atmospheric circulation patterns associated with high-risk daily aerospora counts in Pretoria, we used atmospheric circulation data for 01/1993–02/2023 from the hourly ERA5 reanalysis dataset, with a 0.25° (28 km) square grid resolution (Hersbach et al. 2020). Among the available variables, we used geopotential heights (converted from geopotential by division using Earth's gravitational acceleration) for the 500 hPa (zg500; in m) level along with mean sea level pressure (SLP; units converted to hPa) for a surface and mid-tropospheric identification of synoptic-scale weather systems (e.g., troughs, ridges, cyclones and anticyclones) associated with high-risk aerospora days within Pretoria (Alarcón et al. 2023). Before proceeding, it is important to note why SLP, and not the 850 hPa geopotential height field, is used with zg500 for the Circulation Weather Type (CWT) classification (discussed under Sect. 2.4) over South Africa (namely for Pretoria), a country with the majority of landmass at an elevation > 1000 m.asl. Indeed, the 850 hPa level is typically considered the best level to study weather systems that influence South Africa since this level is at a height just above that of the interior plateau (Reason and Smart 2015). However, in classifying CWTs over South Africa, numerous studies apply SLP (e.g., Ibebuchi 2021; Ireland et al. 2024) as it is can provide a good representation of synoptic-scale systems influencing a region and it can explain the relationship between topography and low-level flow. Furthermore, the South African Weather Service (SAWS) uses SLP to provide daily synoptic charts, and this is the resource we want medical professionals and allergy sufferers to use to diagnose if a day may be associated with high aerospora levels; thus, our classification should consider SLP for this resource to be helpful.

Within the ERA5 data period, we used data for 1993–2022 to assess the atmospheric circulation patterns climatology (for a recent 30-year period), while data for 08/2019–02/2023 were used to explore atmospheric circulation patterns and local-scale meteorological conditions associated with the aerospora sampling period, and particularly for the October-May months during which high-risk days occurred (see Sect. 3.1). To link the large-scale atmospheric circulation patterns associated with high-risk days with more local-scale conditions over Pretoria, we utilised rainfall (units converted to mm), relative humidity (in %), air temperature (units converted to °C), and near-surface 10 m u- and v-wind (in m.s^−1^) for the ERA5 grid cell occupied by the CSIR volumetric trap. While u- and v-wind records were used to calculate wind speeds and directions for each daily time step, we utilised the Magnus approximation to calculate relative humidity from air and dew point temperatures (Alduchov and Eskridge 1996). For our data preparation, hourly ERA5 variables were aggregated on a daily-scale for analyses.

### Data analysis

From the daily aerospora sample, we first defined high-risk days as days with a daily aerospora count > 90th percentile value (i.e., a count greater than 1109 aerospora grains) of all aerospora counts for 08/2019–02/2023. This approach of identifying high-risk days was used for a location-specific identification of high counts as is typically undertaken to define very hot daily temperatures, for instance (e.g., van der Walt and Fitchett 2021). Although this is a good approach to identify more extreme values of variable, limitations and justification of this approach must be noted. Although this 90th percentile value can be used for comparisons across SAPNET sampling locations, it is important to highlight that it is a relative value (and not a threshold value) that is expected to change with additional years of data. Despite this, considering the Burge scale (a scale delineating allergy risk levels based on aerospora levels), values exceeding the 90th percentile level (i.e., a count > 1109 aerospora grains in Pretoria) would be classified as having a risk level that is high, very high, very high and moderate for the tree, grass, weed and fungal spore groupings for aerospora types, respectively (Burge 1992). Therefore, collectively we can assume that the 90th percentile value corresponds to days that pose a high allergenicity risk, and in turn, the 90th percentile value can be used to diagnose allergenicity risk for Pretoria.

Subsequently, atmospheric circulation patterns corresponding to these high-risk days were assessed using a composite analysis approach considering the deviation of conditions during the high-risk days compared to those of the climatologies (Baltaci et al. 2019; Yin et al. 2019; Wang and Zhang 2020; Jury and Buthelezi 2022). Because this approach is commonly applied to identify structural characteristics of atmospheric circulation patterns associated with certain conditions, such as dry and wet conditions (e.g., Mahlalela et al. 2020), we used it to assess if one distinct surface and mid-level synoptic circulation pattern was evident for the high-risk days. Therefore, for the composite analysis, we compared the average SLP and zg500 daily values for the high-risk days with the corresponding October-May climatologies; only October-May months were considered as these correspond to the months when high-risk days occurred (see Sect. 3.1).

Thereafter, to diagnose a range of representative atmospheric circulation patterns that lead to high-risk aerospora levels, a CWT classification was performed using the SynoptReg R package (Lemus-Canovas et al. 2019). Similar to Serrano-Notivoli et al. (2022) and Rodríguez and Lemus-Canovas (2023) who classified CWTs associated with heatwaves and coldwaves, and tornado events respectively, we applied a Principal Component Analysis (PCA) approach to a temporal (T-mode) matrix of daily average SLP and zg500 (for a domain of 10–50°S and 0–55°E) corresponding to all the days within October-May, being the months during which high-risk days were observed; all days were considered to assess if the high-risk days occurred more frequently for some of the CWTs compared to others. Based on a Scree Test (Cattell 1966; Lemus-Canovas et al. 2019; Supplementary Fig. 1), seven PCs were retained, with an explained variance of 71.5%, which subsequently equated to 14 CWTs occurring during the October-May months within the study period (Rodríguez and Lemus-Canovas 2023). To determine whether any CWT was more frequently associated with high-risk days compared to the overall proportion of high-risk days, we applied a two-proportion z-test, considering statistical significance at alpha levels of 5% and 10%. The null (alternative) hypothesis was that the proportion of high-risk days in the CWT group is greater (smaller) than the overall proportion of high-risk days. Finally, to explore local conditions in Pretoria associated with each CWT, descriptive statistics were calculated for rainfall, relative humidity, air temperature, and wind speed and direction for the ERA5 grid cell occupied by the CSIR volumetric trap.

## Results

### Temporal characteristics of high-risk days within Pretoria

**Fig. 2:**
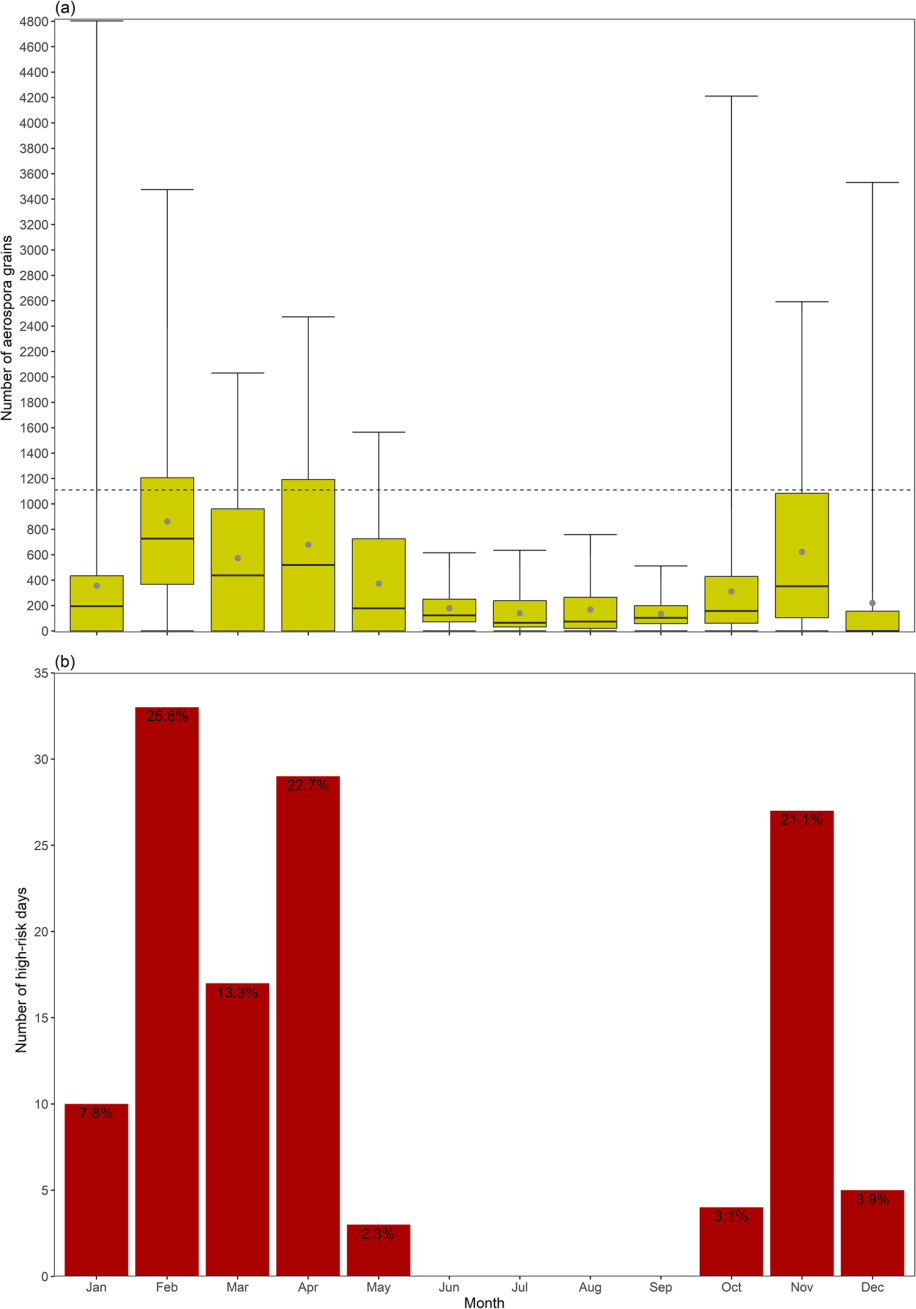
Plot a) shows boxplots depicting the annual cycle of the number of daily aerospora grains grouped by month for 08/2019–02/2023. Grey dots represent the mean number of daily aerospora grains per month, while the whiskers extend to the minimum and maximum number of daily aerospora grains. With respect to the whole dataset, the dashed black line represents the 90th percentile value for the number of daily aerospora grains per month. Plot b) shows the monthly count of high-risk days, being the number of days when the number of daily aerospora grains exceeded the 90th percentile value

For 08/2019–02/2023, the annual aerospora cycle demonstrated a somewhat bimodal distribution, with relatively higher daily average counts during October–November (311–622 aerospora grains on average) and January-May (356–862 aerospora grains on average; Fig. 2a). During June–September, average daily counts were relatively lower, ranging from 134 aerospora grains (September) to 179 aerospora grains (August; Fig. 2a). However, in general, the daily average counts per month ranged from 134 aerospora grains (September) to 862 aerospora grains (February; Fig. 2a). The annual cycle for the high-risk days followed a similar distribution to that for the daily average aerospora cycle, such that high-risk days were only recorded during the October-May months, when many daily aerospora counts were relatively high (Fig. 2a); we, therefore, defined October-May as the aerospora season. Within this aerospora season, a multi-modal distribution was evident for the number of high-risk days, with peaks during November (27 days), February (33 days) and April (29 days) accounting for 69.6% of high-risk days, while overall, a range of three to 33 high-risk days was recorded during May and February respectively (Fig. 2b). Overall, 128 days were detected as days with high-risk aerospora levels during the 08/2019–02/2023 aerospora recording period for Pretoria (Fig. 2b; Supplementary Table 1). The aerospora grain count during these days ranged from 1110 aerospora grains (05–02-2023) to 4803 aerospora grains (28–01-2021; Fig. 2b; Supplementary Table 1).

### Atmospheric circulation patterns associated with high-risk days occurring during October-May within Pretoria

**Fig. 3:**
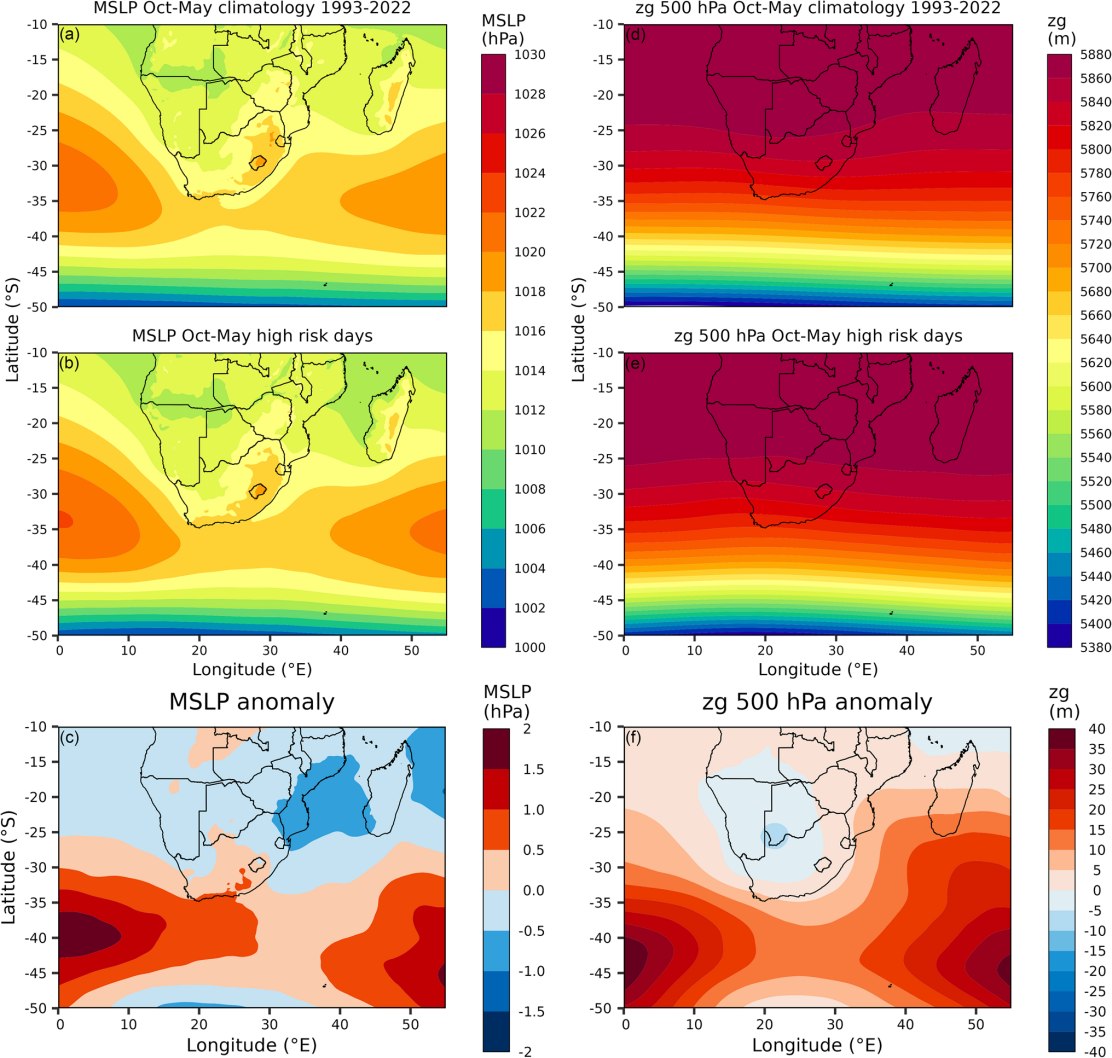
Mean spatial patterns for sea level pressure (SLP) and geopotential heights (zg) at the 500 hPa level (zg500) for the October-May climatology (for 1993–2022; a and d), high-risk days (b and e; see Supplementary Table 1) and anomalies (c and f; i.e., mean of high-risk days minus mean climatology)

Composite analysis results for the SLP and zg500 for the high-risk days within 08/2019–02/2023 compared to the October-May climatology for 1993–2022 demonstrates that no one distinct surface and mid-tropospheric synoptic circulation pattern was associated with the high-risk days (Fig. 3). This is because mean conditions evident for SLP and zg500 for the high-risk days were very similar to the long-term climatologies (compare Fig. 3a to 3b and 3d to 3e), and only minor differences were evident (Fig. 3c, 3f). Therefore, like the vast range of circulation configurations that contributed to characterising the October-May climatology, a range of circulation configurations likely contributed to characterising the mean patterns evident for the high-risk days. Despite this, it is notable that mid-level zg500 conditions overhead of Pretoria (at ~ 25.7°S and ~ 28.2°E) during the high-risk days were up to 5 m above the climatology values (Fig. 3f). For Pretoria, this anomaly indicates that stronger than normal mid-level high-pressure conditions occurred during most (if not all) of the high-risk days, suggesting that these days were characterised by more stable, calm and stagnant air masses which likely hindered dispersion and promoted the accumulation of aerospora.

**Fig. 4:**
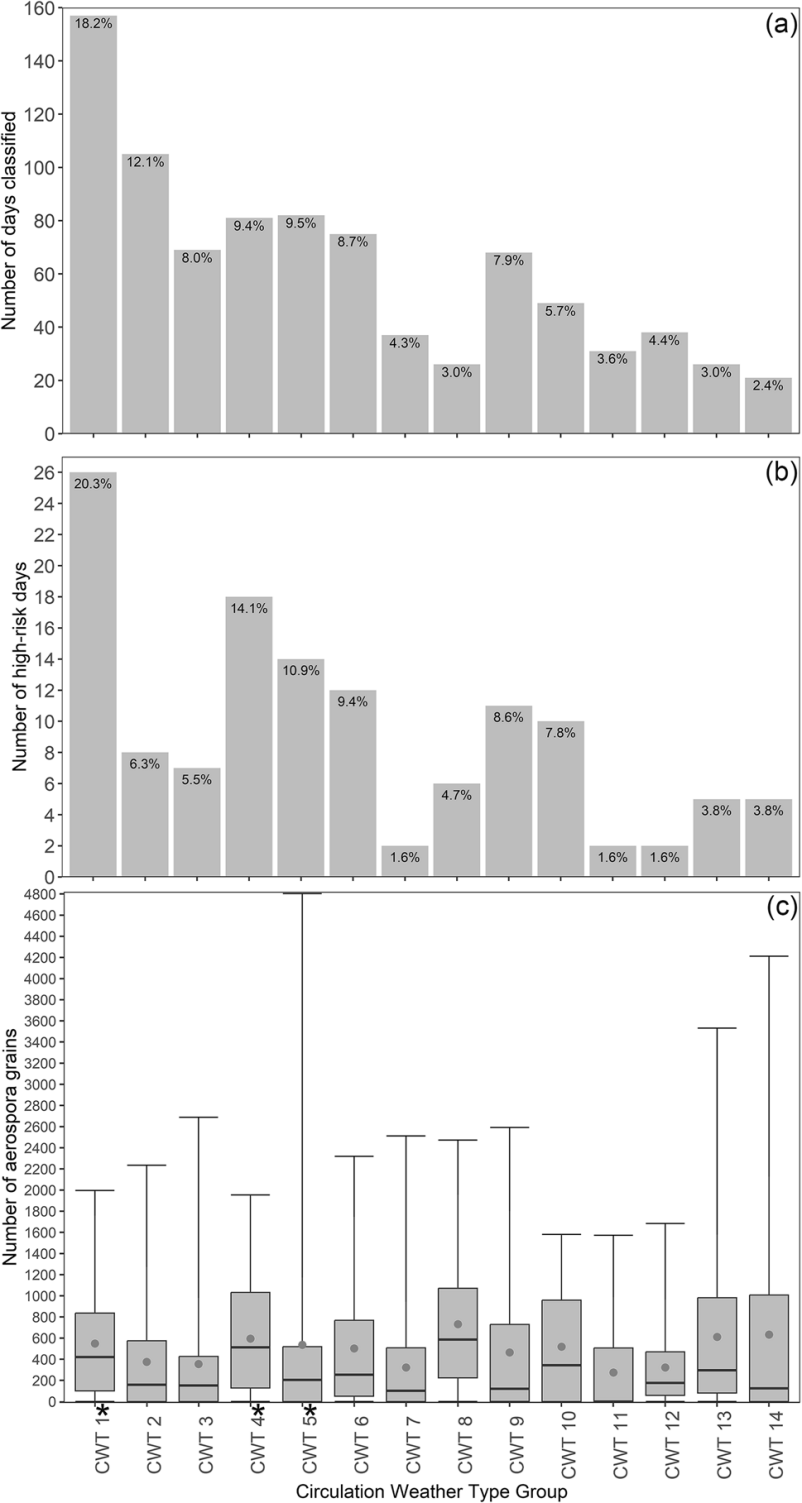
Plot a) shows the daily frequency (number and percentage of days) for each Circulation Weather Type (CWT) group. Plot b) shows the frequency (number and percentage of days) of high-risk days for each CWT. Plot c) shows boxplots depicting the number of daily aerospora grains grouped by CWT. Red dots represent the mean number of daily aerospora grains per CWT, while the whiskers extend to the minimum and maximum number of daily aerospora grains per CWT. An * next to the CWT group label indicates a CWT group with a statistically significant proportion of high-risk days (see Supplementary Table 2 for further information)

Since no distinct surface and/or mid-level circulation pattern was evident for the high-risk days, we classified 14 CWTs, derived from a T-mode PCA approach that classified SLP and zg500 for all days occurring within the October-May months during the study period. All CWT groups were associated with at least two high-risk days, while the highest number of high-risk days within a CWT group was 26 days for CWT 1 (Fig. 4b). Only CWT 1, 4 and 5 were associated with a statistically significant (with p-values of 0.046, 0.035 and 0.077 for CWT 1, 4 and 5, respectively) proportion of high-risk days (Supplementary Table 2). Although CWT 1, 4 and 5 only accounted for 37.1% of classified days (i.e., 320 days overall; Fig. 4a) and only 45.3% of the high-risk days (i.e., 58 high-risk days), it must be noted that our results from here mainly focus on these CWTs, isolating these as the CWTs that had the highest allergenicity risk during the study period October-May months. This is because our work aimed to identify circulation patterns with a statistically significant proportion of days with aerospora grain counts that may pose a high allergenicity risk. For CWT 1, 4 and 5, a respective count of 26 (20.3%), 18 (14,1%) and 14 (10.9%) high-risk days occurred (Fig. 4b). On average, the associated daily aerospora grain count was 548, 594 and 536 for CWT 1, 4 and 5 respectively, whereas the respective maximum daily aerospora grain count recorded was 1995, 1953 and 4803 (Fig. 4c). Notably, compared to other CWT groups, these groups were not consistently associated with the highest average and maximum daily aerospora grain count recorded, and although CWT 5 had the highest maximum aerospora grain count value, CWT 8 and 13–14 had higher average aerospora grain count values of 731, 611 and 632, respectively (Fig. 4c).

**Fig. 5:**
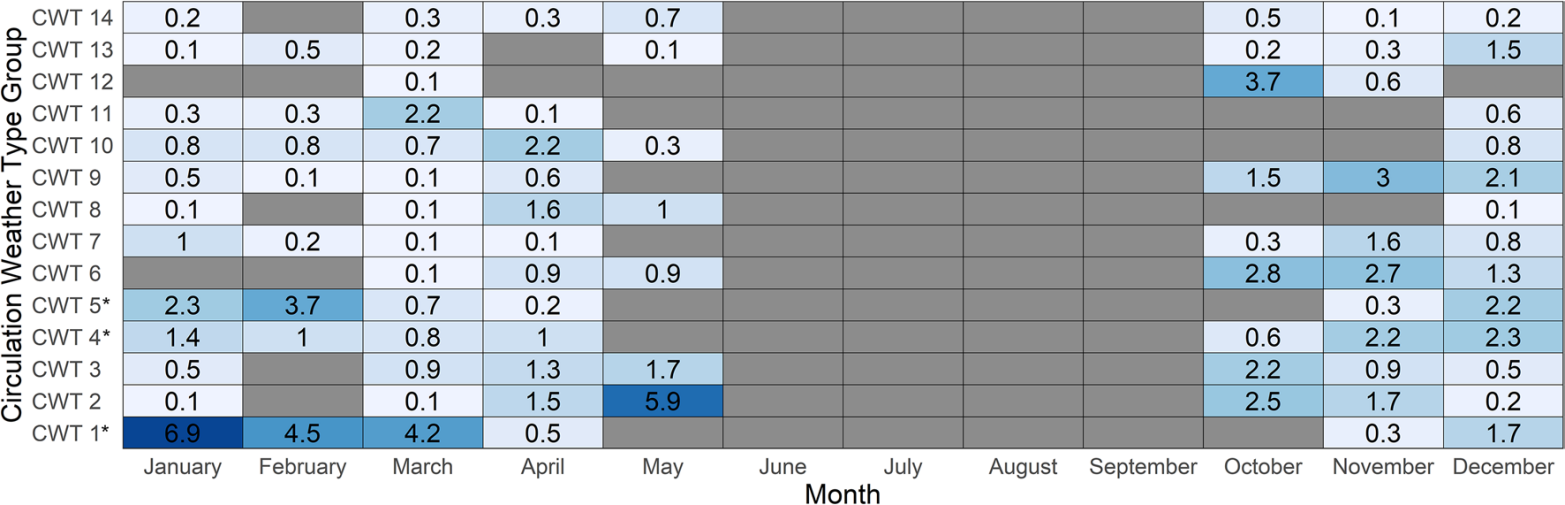
The monthly relative frequency (%) of each of the Circulation Weather Types (CWTs). Note that the CWT monthly relative frequency was only considered for October-May and not June–September. Each monthly relative frequency value was calculated as the total number of days per CWT per month expressed as a percentage of the total number of days in the study period October-May months within 08/2019–02/2023. An * next to the CWT group label indicates a CWT group with a statistically significant proportion of high-risk days (see Supplementary Table 2 for further information)

Temporally, the monthly frequency of all CWTs was heterogeneous (Fig. 5). Collectively, the CWTs associated with the highest risk in terms of high aerospora levels (i.e., CWT 1, 4 and 5) occurred throughout October–April, with CWT 1 and 5 not occurring during October (Fig. 5). The frequency (i.e., number of daily occurrences) of CWT 1 was highest in January (6.9%), while for CWT 4 and 5 the highest frequencies were recorded in November–December (2.2–2.3%) and February (3.7%; Fig. 5). Linking to the annual cycle of high-risk days, it should be highlighted that the occurrence of CWT 1 and 5 likely contributed most to the high frequency of high-risk days evident during February, while CWT 10 and CWT 6 and 4 were characterised by the highest frequency of occurrence for April and November respectively, being months with the second and third highest number of high-risk days (Fig. 2b, 5). Importantly, this result highlights that although the statistically significant proportion of high-risk days for CWT 1, 4 and 5 suggests that these were probably the CWTs associated with the greatest allergenicity risk, they were not the only CWTs associated with allergenicity risk.

**Fig. 6:**
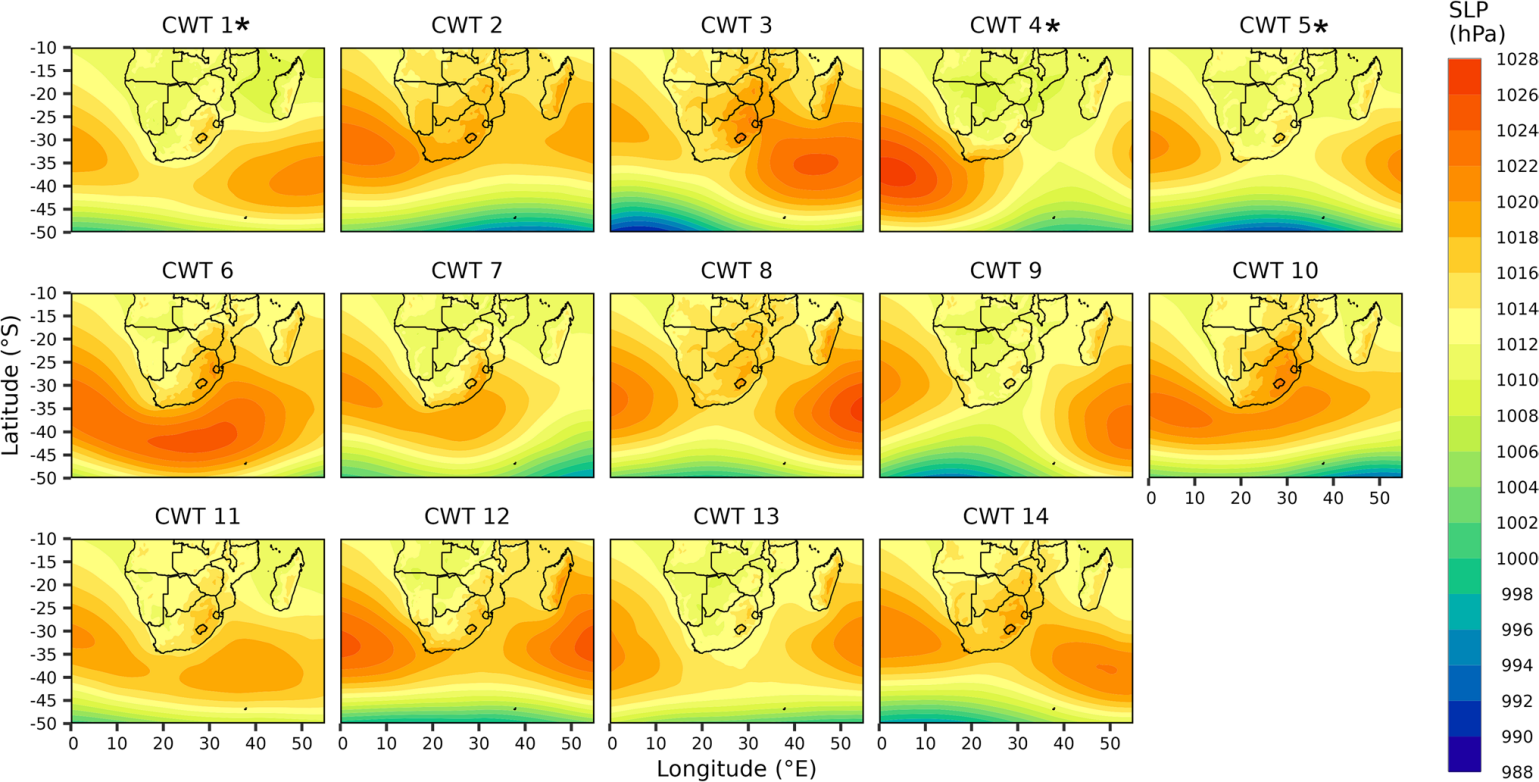
Sea level pressure (SLP) for each Circulation Weather Type (CWT) defined for the October-May days within 08/2019–02/2023 (see Supplementary Table 1). An * next to the CWT group label indicates a CWT group with a statistically significant proportion of high-risk days (see Supplementary Table 2 for further information)

**Fig. 7:**
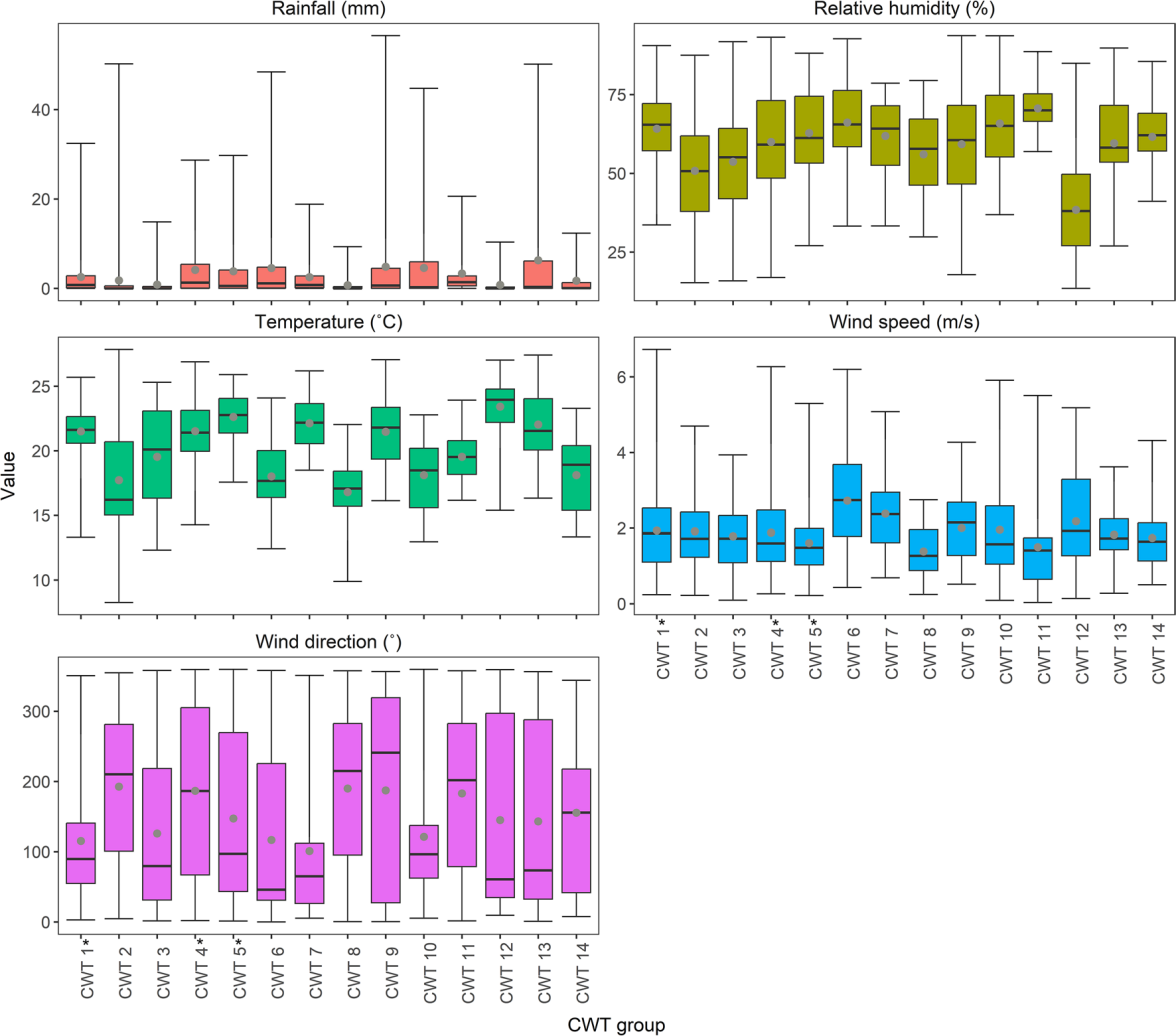
Boxplots depicting the spread of daily rainfall (mm), temperature (°C), relative humidity (%), and wind speed (m.s^−1^) and direction (°) values occurring per Circulation Weather Type (CWT) at the Council for Scientific and Industrial Research (CSIR) sampling location. An * next to the CWT group label indicates a CWT group with a statistically significant proportion of high-risk days (see Supplementary Table 2 for further information)

**Fig. 8:**
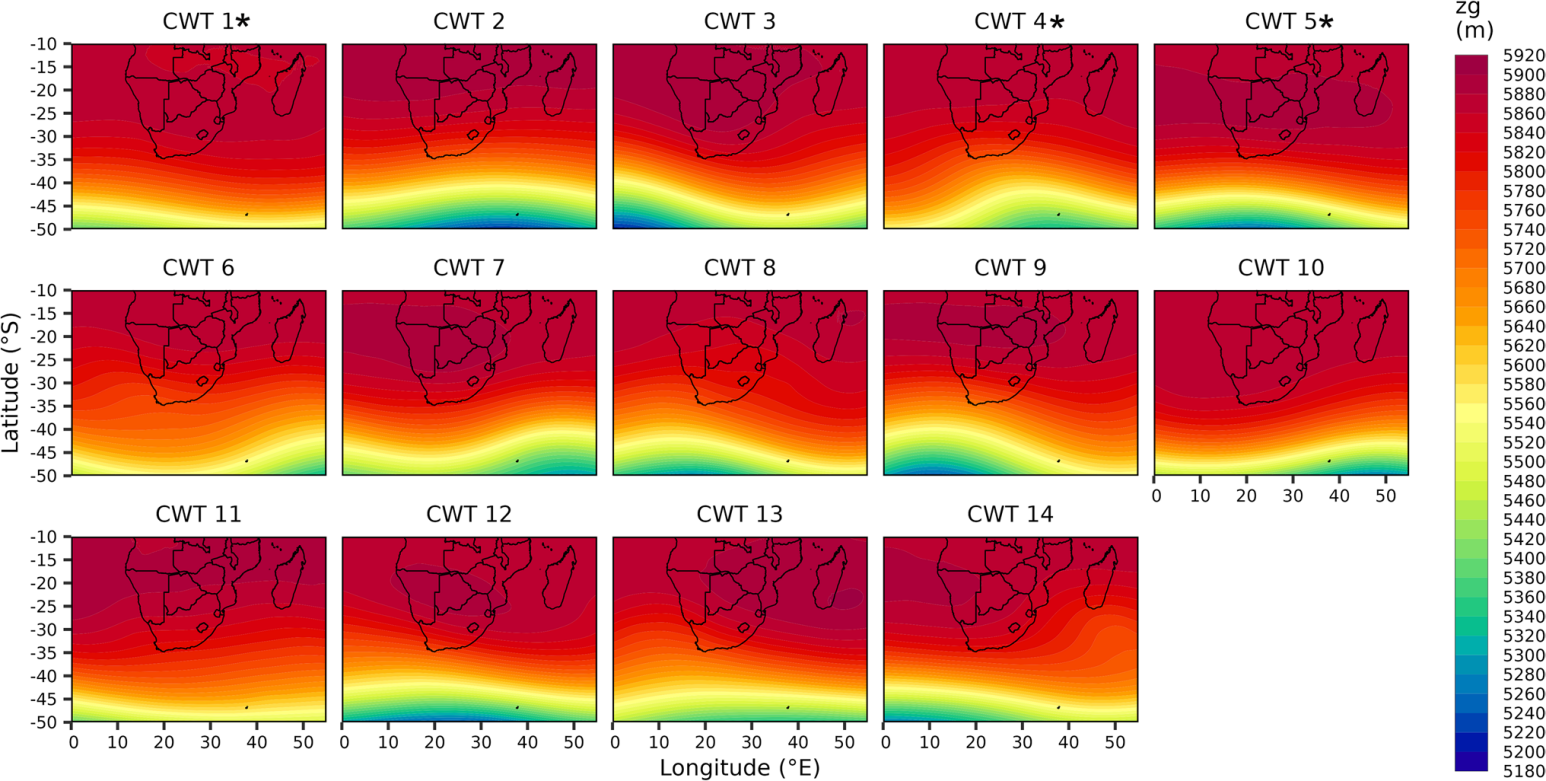
Geopotential heights at the 500 hPa level (zg500) for each Circulation Weather Type (CWT) defined for the October-May days 08/2019–02/2023 (see Supplementary Table 1). An * next to the CWT group label indicates a CWT group with a statistically significant proportion of high-risk days (see Supplementary Table 2 for further information)

Nonetheless, towards forecasting and assessing allergenicity risk on a given day, it is valuable to understand the large-scale surface and mid-tropospheric circulation patterns and associated local-scale (i.e., for Pretoria) meteorological conditions for CWT 1, 4 and 5, being the CWT groups more frequently associated with high-risk days. Among these CWTs, CWT 1 and 5 demonstrated the most similar conditions, however, they were still distinctly different (Fig. 6–8); notably CWT 9 and 12 are also characterised by relatively similar large-scale and local-scale patterns to CWT 1 and 5. At the surface, the SLP configuration for CWT 1 and 5 was a pattern of anticyclonic high-pressure systems located adjacent to the west and east of South Africa (Fig. 6). Among these and particularly during these CWTs, the South Indian Anticyclone (SIA), to the east and extending over eastern parts of the country (to regions including Pretoria), was probably the most influential in terms of limiting aerospora dispersion/deposition and promoting accumulation that occurred over Pretoria when these CWTs prevailed (Fig. 6). This is because this extension inland over central-eastern regions of South Africa promoted slight high-pressure conditions (up to 1016 hPa over Pretoria; Fig. 6), which were associated with relatively warm (average temperatures of 21.5 and 22.6 ˚C), dry (average rainfall of 2.5 and 3.8 mm with an average relative humidity of 64.1 and 62.8%) and stagnant (predominantly easterly winds with average wind speeds of 1.9 and 1.6 m.s^−1^) daily conditions (Fig. 7; Supplementary Fig. 3); such conditions can favor aerospora accumulation. Moving aloft, the fact that the mid-tropospheric Botswana High (BH) was well established over the interior Highveld regions of southern Africa for the CWT 1 and 5 patterns highlights that mid-tropospheric circulation configuration was also very influential in terms of promoting accumulation of aerospora over Pretoria (Fig. 8).

CWT 4 differed from CWT 1 and 5 in that the surface South Atlantic Anticyclone (SAA) was extended east over western South Africa, while inland over most central regions of South Africa a trough extending southward was prevalent (Fig. 6). When CWT 4 occurred, the average pattern depicted that relatively low-pressure conditions, up to 1012 hPa, occurred over Pretoria, and were accompanied by relatively slow westerly to northerly winds (averaging 3 m.s^−1^), relatively wetter conditions (average rainfall of 4.3 mm with average relative humidity of 59.9%) and relatively warm temperatures (average temperatures of 21.5 ˚C; Fig. 7). Aloft, at the mid-tropospheric level, a trough was also evident, extending north to central South Africa (Fig. 8). Compared to high-pressure conditions, surface and mid-tropospheric troughs may be considered less likely to promote the accumulation of aerospora for a given location. However, for CWT 4, the warmer, more humid and relatively wet conditions could promote the release of aerospora within Pretoria.

## Discussion, conclusions, and recommendations

Within the period of 08/2019–02/2023, this study focused on the October-May months which were characterised by the occurrence of at least three high-risk days per month (Fig. 2b), when daily aerospora grain counts were likely to pose a high allergenicity risk, potentially triggering allergic reactions for sensitive individuals within Pretoria. For these months, our results showed that high-risk days were more typically associated with above-normal mid-tropospheric geopotential heights over the Pretoria region (Fig. 3f); however, no single atmospheric circulation configuration could be defined for high-risk days. Therefore, we developed a CWT classification of 14 CWTs (Fig. 6, 8), derived from daily average SLP and zg500 fields of the ERA5 reanalysis dataset, and following the application of a two-proportion z-test, we found that three CWTs were more frequently associated with high-risk days. Similar to the study of CWTs and those associated with heatwaves over South Africa (Ireland et al. 2023), we argue that our results of these CWTs which are associated with the greatest risk in terms of high aerospora levels are most meaningful if they can be considered in the context of early warning to alert vulnerable groups, such as the allergic population within Pretoria.

To understand and contextualise the risk associated with the high-risk days we have isolated, it is necessary to consider our definition of high-risk days in the context of the high-risk season and the aerospora types recorded during these days. Considering previous research regarding aerospora over Pretoria, it is notable that the October-May high-risk aerospora season defined here is within the September-July season defined for Pretoria by only considering pollen types (Esterhuizen et al. 2023). This confirms that our focus period of October-May was relevant in the context of increased allergenicity risk. A further assessment of risk during these high-risk days requires consideration of aerospora types, and although we leave this as work for future research which could link CWTs and specific aerospora types, we briefly provide information about this in the supplementary material to aid in discussing our results. Similar to the study for Johannesburg (~ 60 km south of Pretoria) by Ajikah et al. (2023), we found that fungal spores had higher grain counts from approximately January-May (and more specifically up to July for the Johannesburg study) and during October, and fungal spores were also characterised by the highest counts in the Pretoria sample, both overall and for the high-risk days (Supplementary Fig. 3a; Supplementary Table 1); thus, the high-risk aerospora season we identified pinpointed the time period when fungal spores were most likely to cause significant allergic reactions. These are key points to note because fungal spores are typically found at high levels in aerobiological monitoring, and consequently they are important allergens (Grinn-Gofroń et al. 2020). Another similarity to the Johannesburg study was that despite these high counts, the fungal spore grain counts detected overall and for the high-risk days were more frequently at low and moderate allergy risk levels according to the Burge scale (Supplementary Table 1; Supplementary Fig. 3a; Burge 1992). Despite this suggested low-moderate risk, research for South Africa suggests that these levels still pose a significant allergenic risk, especially for more sensitive individuals who would likely experience significant symptoms at these levels (Potter et al. 1991; Joubert 2006; SAPNET 2019). Moreover, it has also been found that fungal allergy is a risk factor for South African individuals with more severe asthma, especially for polysensitised individuals (Potter 2010).

To assess the potential impacts experienced by allergic individuals during the high-risk days, it is also necessary to briefly consider the pollen types that likely occurred during these days. Similar to the fungal spores, the grain counts observed for the grass, tree and weed groupings were also only suggested to be of low to moderate risk according to the Burge scale (Supplementary Table 1; Supplementary Fig. 3; Burge 1992). At a low-moderate risk level, a respective level of < 20% and > 50% of pollen allergy sufferers may experience allergic and asthmatic symptoms, so preventative therapies (e.g., nasal steroid sprays) and acute treatments (e.g., non-sedating antihistamines) are recommended (SAPNET 2019). Considering the pollen types likely during high-risk days, we refer to the study by Esterhuizen et al. (2023) which demonstrated that Poaceae (grass), various weeds (including sedges being Cyperaceae, slangbos being *Stoebe-type*, the daisy family being Asteraceae and goosefoot being Chenopodiaceae, among others) and various trees (namely Moraceae being mulberry, Betulaceae being birch, *Platanus* sp. being plane, Ulmaceae being elm family and Myrtaceae being myrtle family), were the most common airborne pollen types occurring in Pretoria. Their results reflected that Poaceae had high concentrations throughout October-July, with peaks during January-March, while tree types had a season generally spanning September-December, with some occurrences during February-May for Myrtaceae (similar to Supplementary Fig. 3b-c; Esterhuizen et al. 2023). For the weed types, higher concentrations were evident during February-April (similar to Supplementary Fig. 3d; Esterhuizen et al. 2023). Observation of these most common pollen types and the period during which higher grain counts (or concentrations) occur is important to confirm that the high-risk period considered herein was indeed a relevant period when pollen levels were higher. Also, all the dominant types noted by Esterhuizen et al. (2023) are suggested to be associated with moderate or major allergenicity risk in a South African context (Cadman 1991; Joubert 2006; Singh and Mathur 2012; van Rooyen et al. 2020). Among these common pollen types, Poaceae is known as a leading aeroallergen worldwide and can cause allergic rhinitis, allergic conjunctivitis, and asthma (García-Mozo 2017). On the other hand, due to limited research, trees are suggested to be an uncommon cause of seasonal allergies in South Africa; however, over short periods, trees can release high pollen quantities, making them problematic for allergy sufferers as high airborne pollens levels can easily trigger pollinosis (Ajikah et al. 2020, 2023; Gharbi et al. 2023). Similarly, weeds are also thought to contribute minor allergic reactions (or pose a slight risk to people with allergies) in South Africa, however, much more research is needed to better understand their allergenicity risk (Ajikah et al. 2020).

Considering the risk associated with the aerospora types commonly occurring over Pretoria, our results isolated three CWTs posing a heightened allergenicity risk, including CWT 1, 4 and 5. To discuss the conditions associated with these CWTs, it is important to highlight that they accounted for only 45.3% of high-risk days (Fig. 4b). The limited aerospora dataset (spanning only three-and-a-half-years, from 08/2019) may have contributed to this finding; thus, with additional years of aerospora data, a more robust classification, similar to Ireland et al. (2024) can be developed for Pretoria as well as other SAPNET monitoring sites. Nonetheless, by isolating three CWTs which were more frequently associated with high-risk days, our study has contributed to furthering our understanding of aerospora meteorological patterns. For Pretoria, this new knowledge can be applied via the SAPNET website to contribute to anticipating and mitigating situations that may compromise the health of populations and for the adoption of prevention measures by competent health authorities.

Each of the CWTs associated with high allergenicity risk were distinct, with some similarities between CWTs 1 and 5, which collectively accounted for 34.4% of high-risk days (Fig. 4b). Over Pretoria, these CWTs were associated with anticyclonic conditions at the surface and mid-troposphere (Fig. 6 and 8), while at the surface, local-scale conditions in a South African context reflected relatively dry, warm, stable and calm conditions (Fig. 7; Tyson and Preston-Whyte 2000). The statistically significant association between high-risk aerospora levels and anticyclonic conditions is therefore not unexpected for the Pretoria region, especially if aerospora are considered similar to air pollutants whose concentrations over the South African Highveld inland plateau region (where Pretoria is located) are often higher when anticyclonic circulation types prevail over much of eastern and central South Africa (Jury 2017; Matandirotya and Burger 2021, 2023; Lai et al. 2023). Elsewhere, including Poland, the Iberian Peninsula and Greece, studies similarly demonstrate that aerospora concentrations are typically higher when prevailing conditions are anticyclonic and associated with a stable atmosphere, inducing dry, calm, and warm conditions (Ojrzyńska et al. 2020; Paschalidou et al. 2020; Alarcón et al. 2023). Over Poland, Ojrzyńska et al. (2020) demonstrated that anticyclonic conditions promote high pollen concentrations regardless of the pollen taxon considered. Interestingly, over and above the allergenicity risks highlighted, CWT 1 and 5 are also very similar to CWT 1 and 4 presented in Ireland et al. (2024), who demonstrated an association with high heat-related health risks. This highlights that anticyclonic circulation patterns occurring over South Africa’s Highveld region are closely linked to health-related risks associated with air pollution, heatwaves and aerospora. For CWT 4 herein, we also found a statistically significant proportion of high-risk days, which was surprising because research for other parts of the world suggests that relatively wet, humid and warm low-pressure conditions are not frequently associated with high airborne pollen levels (Ojrzyńska et al. 2020; Paschalidou et al. 2020; Alarcón et al. 2023). Such conditions can reduce airborne pollen levels, but studies have found that higher relative humidity levels, in particular, may promote higher airborne fungal spore levels (Grinn-Gofroń et al. 2020; Ajikah et al. 2023). This finding underscores the importance of further research considering CWTs and their links to specific aerospora types. We suggest this as a future research topic for all SAPNET monitoring sites, considering aerospora types individually since Esterhuizen et al. (2023) demonstrate significant variation in pollen seasons across the SAPNET monitoring locations.

Moving forward, we conclude that our study has contributed to furthering our understanding of aerospora meteorological patterns. For Pretoria, this new knowledge can be applied to anticipate situations that may compromise the health of populations and for the adoption of prevention measures by competent health authorities. Our study offers valuable insights for other SAPNET monitoring sites and for regions with existing aerospora monitoring but limited early warning capabilities, as our study can aid in contributing to the development of practical methods for creating early warning systems that will benefit allergy sufferers. Over Pretoria, our findings highlight that within the October-May high-risk period, November, February and April are months characterised by particularly high risk; thus, health authorities must be on high alert during October-May, especially during November, February and April. Together with South Africa’s meteorological agency (i.e., SAWS), the SAPNET monitoring team should develop an early warning system that can be based on already available forecast information, such as from Windy or from organisations such as SAWS or the Agricultural Research Council that are already known to provide forecast information for South Africa. This information can be published on the SAPNET website to issue alerts when conditions reflecting CWTs 1, 4 and 5 occur for Pretoria; the same can be done for other SAPNET sites once similar studies have been undertaken. Therefore, on these days, advisories targeted at sensitive individuals in Pretoria could recommend that they stay indoors, use air purifiers, and take preventive medications. To complement the results herein, further research can consider statistical forecasting for individual SAPNET monitoring locations using deep learning algorithms, similar to Pillay et al. (2023). This will strengthen the practical application of our findings, which is to inform early warning systems. By providing timely advisories, we can empower sensitive individuals, such as allergy sufferers, to take necessary precautions during high-risk periods.

## Supplementary Information

Below is the link to the electronic supplementary material.

Supplementary file1 (DOCX 4850 KB)

## Data Availability

The aeropalynological dataset used herein is available on request from the South African Pollen-monitoring Network (SAPNET; https://pollencount.co.za/). All of the ERA5 data used herein are freely available for download from the Copernicus Climate Data store (https://cds.climate.copernicus.eu/#!/home).
