# Supplementary material for: High aerospora levels and associated atmospheric circulation patterns: Pretoria, South Africa
Source: Int J Biometeorol. 2024 Sep 28;69(10):2441–56. doi: 10.1007/s00484-024-02781-8 (PMC12540555; doi:10.1007/s00484-024-02781-8)
Supplement: Supplementary file 1 — Supplementary file1 (DOCX 4850 KB) [file 484_2024_2781_MOESM1_ESM.docx]

# **Supplementary information for: High aerospora levels and associated atmospheric circulation patterns: Pretoria, South Africa**

Roffe SJ^*1,2,3^, Ajikah LB^4,5,6^, John J^7^, Garland RM^8^, Lehtipalo K^6^, Bamford MK^4^

Corresponding author^*^: [RoffeS@arc.agric.za](mailto:RoffeS@arc.agric.za)

^1^Agrometeorology Division, Agricultural Research Council - Natural Resources and Engineering, Pretoria, South Africa

^2^Department of Geography, University of the Free State, Bloemfontein, South Africa

^3^Global Change Institute, University of the Witwatersrand, Johannesburg, South Africa

^4^Evolutionary Studies Institute, University of the Witwatersrand, Johannesburg, South Africa

^5^Department of Botany, University of Calabar, Calabar, Nigeria

^6^Institute of Atmospheric and Earth System Research, University of Helsinki, Helsinki, Finland

^7^SMART Places, Council for Scientific and Industrial Research, Pretoria, South Africa

^8^Department of Geography, Geoinformatics and Meteorology, University of Pretoria, Pretoria, South Africa


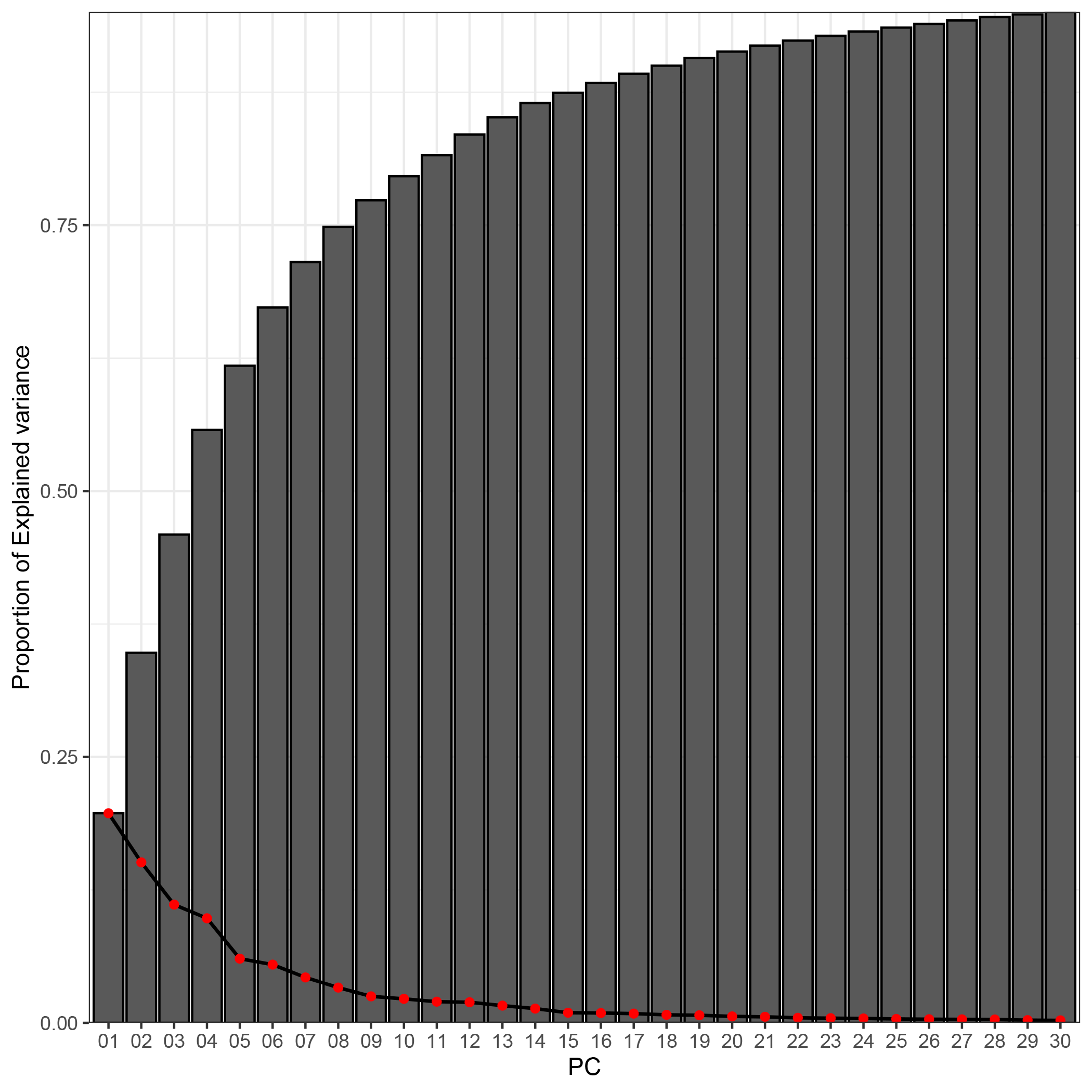


**Supplementary Figure 1:** The explained variance of each principal component (PC) for the Principal Component Analysis (PCA) applied to classify Circulation Weather Types (CWTs) for Pretoria. The grey bars represent cumulative variance, and the red dots represent the proportion of explained variance for each PC. Based on an accumulated variance of 71.5%, seven PCs were selected, resulting in 14 CWTs. This selected cumulative variance amount is based on guidance from previous studies having typically selected PCs based on an explained cumulative variance of ~60-75% (Serrano-Notivoli et al., 2022; Rodríguez and Lemus-Canovas 2023).

**Supplementary Table 1:** Daily count (as number of aerospora grains) of fungal spores (as number of fungal spore grains), grass pollen (as number of grass pollen grains), tree pollen (as number of tree pollen grains) and weed pollen (as number of weed pollen grains) and associated Circulation Weather Types (CWT) groups for days with aerospora grain counts above the 90th percentile (>1109 aerospora grains).

| Date | Fungal spores (count) | Fungal spores (%) | Grasses (count) | Grasses (%) | Trees (count) | Trees (%) | Weeds  (count) | Weeds  (%) | Daily aerospora count | CWT group |
| --- | --- | --- | --- | --- | --- | --- | --- | --- | --- | --- |
|  |  |  |  |  |  |  |  |  |  |  |
| 2020/02/10 | 1335 | 96.5 | 47 | 3.4 | 1 | 0.1 | 0 | 0 | 1383 | 11 |
| 2020/02/14 | 999 | 89.1 | 60 | 5.4 | 6 | 0.5 | 56 | 5 | 1121 | 13 |
| 2020/02/15 | 1216 | 94.6 | 67 | 5.2 | 3 | 0.2 | 0 | 0 | 1286 | 13 |
| 2020/02/17 | 1089 | 92.9 | 51 | 4.4 | 28 | 2.4 | 4 | 0.3 | 1172 | 5 |
| 2020/10/06 | 4209 | 100 | 0 | 0 | 1 | 0 | 1 | 0 | 4211 | 14 |
| 2020/10/07 | 1262 | 99.4 | 1 | 0.1 | 6 | 0.5 | 1 | 0.1 | 1270 | 14 |
| 2020/10/09 | 1378 | 99 | 2 | 0.1 | 8 | 0.6 | 4 | 0.3 | 1392 | 4 |
| 2020/11/10 | 1143 | 100 | 0 | 0 | 0 | 0 | 0 | 0 | 1143 | 4 |
| 2020/11/11 | 2498 | 99.5 | 0 | 0 | 13 | 0.5 | 0 | 0 | 2511 | 7 |
| 2021/01/16 | 1581 | 98.1 | 29 | 1.8 | 2 | 0.1 | 0 | 0 | 1612 | 1 |
| 2021/01/25 | 1282 | 99.9 | 1 | 0.1 | 0 | 0 | 0 | 0 | 1283 | 1 |
| 2021/01/26 | 1758 | 100 | 0 | 0 | 0 | 0 | 0 | 0 | 1758 | 1 |
| 2021/01/27 | 1886 | 100 | 0 | 0 | 0 | 0 | 0 | 0 | 1886 | 5 |
| 2021/01/28 | 4803 | 100 | 0 | 0 | 0 | 0 | 0 | 0 | 4803 | 5 |
| 2021/02/01 | 1234 | 99.8 | 1 | 0.1 | 1 | 0.1 | 0 | 0 | 1236 | 13 |
| 2021/02/03 | 2383 | 99.8 | 1 | 0 | 2 | 0.1 | 1 | 0 | 2387 | 5 |
| 2021/02/04 | 2164 | 99.6 | 0 | 0 | 0 | 0 | 8 | 0.4 | 2172 | 5 |
| 2021/02/05 | 2056 | 100 | 0 | 0 | 0 | 0 | 0 | 0 | 2056 | 5 |
| 2021/02/06 | 3471 | 99.9 | 3 | 0.1 | 0 | 0 | 0 | 0 | 3474 | 5 |
| 2021/02/24 | 1107 | 99.4 | 6 | 0.5 | 0 | 0 | 1 | 0.1 | 1114 | 4 |
| 2021/02/26 | 1128 | 99 | 10 | 0.9 | 1 | 0.1 | 0 | 0 | 1139 | 4 |
| 2021/02/27 | 1560 | 98.7 | 12 | 0.8 | 3 | 0.2 | 5 | 0.3 | 1580 | 10 |
| 2021/03/31 | 1725 | 97.2 | 15 | 0.8 | 28 | 1.6 | 7 | 0.4 | 1775 | 2 |
| 2021/04/01 | 1884 | 97.5 | 20 | 1 | 5 | 0.3 | 24 | 1.2 | 1933 | 2 |
| 2021/04/02 | 2205 | 98.7 | 20 | 0.9 | 4 | 0.2 | 6 | 0.3 | 2235 | 2 |
| 2021/04/07 | 1323 | 99.6 | 2 | 0.2 | 1 | 0.1 | 2 | 0.2 | 1328 | 3 |
| 2021/04/08 | 1507 | 99.1 | 8 | 0.5 | 0 | 0 | 5 | 0.3 | 1520 | 14 |
| 2021/04/14 | 1182 | 97.1 | 21 | 1.7 | 0 | 0 | 14 | 1.2 | 1217 | 10 |
| 2021/04/30 | 1134 | 97 | 18 | 1.5 | 7 | 0.6 | 10 | 0.9 | 1169 | 4 |
| 2021/11/05 | 1671 | 98.8 | 3 | 0.2 | 16 | 0.9 | 1 | 0.1 | 1691 | 2 |
| 2021/11/06 | 1752 | 97.3 | 7 | 0.4 | 33 | 1.8 | 8 | 0.4 | 1800 | 9 |
| 2021/11/07 | 1557 | 99 | 4 | 0.3 | 10 | 0.6 | 1 | 0.1 | 1572 | 9 |
| 2021/11/08 | 2295 | 99.8 | 2 | 0.1 | 3 | 0.1 | 0 | 0 | 2300 | 9 |
| 2021/11/20 | 1152 | 99.7 | 1 | 0.1 | 2 | 0.2 | 1 | 0.1 | 1156 | 6 |
| 2021/11/22 | 1989 | 99.4 | 6 | 0.3 | 3 | 0.1 | 3 | 0.1 | 2001 | 6 |
| 2021/11/26 | 1539 | 99.8 | 1 | 0.1 | 1 | 0.1 | 1 | 0.1 | 1542 | 6 |
| 2021/11/29 | 1182 | 98.6 | 3 | 0.3 | 12 | 1 | 2 | 0.2 | 1199 | 9 |
| 2021/12/06 | 3516 | 99.6 | 5 | 0.1 | 2 | 0.1 | 8 | 0.2 | 3531 | 13 |
| 2021/12/08 | 1254 | 98.6 | 3 | 0.2 | 6 | 0.5 | 9 | 0.7 | 1272 | 7 |
| 2021/12/10 | 2661 | 99 | 10 | 0.4 | 12 | 0.4 | 5 | 0.2 | 2688 | 3 |
| 2022/01/17 | 1182 | 97.1 | 33 | 2.7 | 2 | 0.2 | 0 | 0 | 1217 | 1 |
| 2022/01/18 | 1430 | 97.8 | 21 | 1.4 | 10 | 0.7 | 1 | 0.1 | 1462 | 14 |
| 2022/01/19 | 2077 | 97.6 | 32 | 1.5 | 13 | 0.6 | 5 | 0.2 | 2127 | 14 |
| 2022/01/27 | 1121 | 95.2 | 55 | 4.7 | 1 | 0.1 | 1 | 0.1 | 1178 | 1 |
| 2022/02/01 | 1392 | 95 | 60 | 4.1 | 9 | 0.6 | 4 | 0.3 | 1465 | 1 |
| 2022/02/02 | 1854 | 94.7 | 90 | 4.6 | 10 | 0.5 | 3 | 0.2 | 1957 | 1 |
| 2022/02/03 | 1734 | 95.4 | 62 | 3.4 | 19 | 1 | 2 | 0.1 | 1817 | 5 |
| 2022/02/05 | 1048 | 91.4 | 91 | 7.9 | 4 | 0.3 | 3 | 0.3 | 1146 | 1 |
| 2022/02/06 | 1004 | 89.7 | 106 | 9.5 | 4 | 0.4 | 5 | 0.4 | 1119 | 1 |
| 2022/02/08 | 1290 | 97.5 | 10 | 0.8 | 1 | 0.1 | 22 | 1.7 | 1323 | 1 |
| 2022/02/09 | 2531 | 98 | 24 | 0.9 | 9 | 0.3 | 18 | 0.7 | 2582 | 5 |
| 2022/02/10 | 1404 | 95.5 | 43 | 2.9 | 3 | 0.2 | 20 | 1.4 | 1470 | 5 |
| 2022/02/11 | 1633 | 96.3 | 36 | 2.1 | 15 | 0.9 | 12 | 0.7 | 1696 | 5 |
| 2022/02/12 | 1231 | 97.3 | 26 | 2.1 | 6 | 0.5 | 2 | 0.2 | 1265 | 10 |
| 2022/03/01 | 1167 | 99.5 | 4 | 0.3 | 2 | 0.2 | 0 | 0 | 1173 | 1 |
| 2022/03/10 | 1314 | 98.6 | 16 | 1.2 | 0 | 0 | 2 | 0.2 | 1332 | 1 |
| 2022/03/17 | 1473 | 97.9 | 9 | 0.6 | 9 | 0.6 | 14 | 0.9 | 1505 | 1 |
| 2022/03/18 | 1398 | 98.3 | 7 | 0.5 | 8 | 0.6 | 9 | 0.6 | 1422 | 1 |
| 2022/03/19 | 1431 | 99.4 | 6 | 0.4 | 2 | 0.1 | 0 | 0 | 1439 | 10 |
| 2022/03/20 | 1121 | 97.6 | 18 | 1.6 | 5 | 0.4 | 5 | 0.4 | 1149 | 10 |
| 2022/03/21 | 1281 | 99.4 | 6 | 0.5 | 2 | 0.2 | 0 | 0 | 1289 | 10 |
| 2022/03/22 | 1359 | 98.8 | 7 | 0.5 | 1 | 0.1 | 9 | 0.7 | 1376 | 10 |
| 2022/03/23 | 1567 | 99.2 | 10 | 0.6 | 1 | 0.1 | 1 | 0.1 | 1579 | 13 |
| 2022/03/24 | 1728 | 99.4 | 8 | 0.5 | 0 | 0 | 2 | 0.1 | 1738 | 4 |
| 2022/03/25 | 1644 | 99.6 | 5 | 0.3 | 0 | 0 | 2 | 0.1 | 1651 | 4 |
| 2022/03/26 | 1560 | 99.2 | 4 | 0.3 | 0 | 0 | 8 | 0.5 | 1572 | 11 |
| 2022/03/28 | 2016 | 99.3 | 3 | 0.1 | 2 | 0.1 | 9 | 0.4 | 2030 | 3 |
| 2022/03/29 | 1857 | 99.1 | 4 | 0.2 | 3 | 0.2 | 10 | 0.5 | 1874 | 3 |
| 2022/03/30 | 1920 | 98.3 | 15 | 0.8 | 3 | 0.2 | 16 | 0.8 | 1954 | 8 |
| 2022/03/31 | 1665 | 98.9 | 12 | 0.7 | 1 | 0.1 | 5 | 0.3 | 1683 | 12 |
| 2022/04/01 | 1269 | 96.8 | 17 | 1.3 | 5 | 0.4 | 20 | 1.5 | 1311 | 4 |
| 2022/04/02 | 1239 | 98.4 | 10 | 0.8 | 8 | 0.6 | 2 | 0.2 | 1259 | 4 |
| 2022/04/06 | 1635 | 99.2 | 2 | 0.1 | 6 | 0.4 | 6 | 0.4 | 1649 | 4 |
| 2022/04/07 | 1141 | 96.4 | 8 | 0.7 | 10 | 0.8 | 25 | 2.1 | 1184 | 4 |
| 2022/04/08 | 1419 | 98.1 | 9 | 0.6 | 4 | 0.3 | 15 | 1 | 1447 | 6 |
| 2022/04/09 | 2292 | 98.8 | 8 | 0.3 | 7 | 0.3 | 12 | 0.5 | 2319 | 6 |
| 2022/04/10 | 1641 | 99.2 | 7 | 0.4 | 3 | 0.2 | 3 | 0.2 | 1654 | 1 |
| 2022/04/11 | 1492 | 98.8 | 9 | 0.6 | 2 | 0.1 | 7 | 0.5 | 1510 | 1 |
| 2022/04/12 | 1638 | 98.8 | 8 | 0.5 | 0 | 0 | 12 | 0.7 | 1658 | 1 |
| 2022/04/13 | 1623 | 99 | 6 | 0.4 | 6 | 0.4 | 5 | 0.3 | 1640 | 1 |
| 2022/04/14 | 2457 | 99.4 | 5 | 0.2 | 2 | 0.1 | 8 | 0.3 | 2472 | 8 |
| 2022/04/15 | 1947 | 99.7 | 1 | 0.1 | 1 | 0.1 | 4 | 0.2 | 1953 | 4 |
| 2022/04/16 | 1127 | 99.9 | 1 | 0.1 | 0 | 0 | 0 | 0 | 1128 | 10 |
| 2022/04/18 | 1173 | 98.2 | 5 | 0.4 | 6 | 0.5 | 10 | 0.8 | 1194 | 10 |
| 2022/04/19 | 1164 | 99.2 | 3 | 0.3 | 1 | 0.1 | 5 | 0.4 | 1173 | 10 |
| 2022/04/20 | 1224 | 98.5 | 9 | 0.7 | 1 | 0.1 | 9 | 0.7 | 1243 | 3 |
| 2022/04/21 | 1461 | 98.8 | 6 | 0.4 | 1 | 0.1 | 10 | 0.7 | 1478 | 8 |
| 2022/04/22 | 1296 | 99.2 | 5 | 0.4 | 1 | 0.1 | 5 | 0.4 | 1307 | 8 |
| 2022/04/23 | 1923 | 99.6 | 5 | 0.3 | 1 | 0.1 | 1 | 0.1 | 1930 | 8 |
| 2022/04/25 | 1131 | 99.7 | 1 | 0.1 | 1 | 0.1 | 1 | 0.1 | 1134 | 4 |
| 2022/04/26 | 1089 | 97.7 | 3 | 0.3 | 3 | 0.3 | 20 | 1.8 | 1115 | 4 |
| 2022/04/29 | 1572 | 98.4 | 11 | 0.7 | 2 | 0.1 | 13 | 0.8 | 1598 | 2 |
| 2022/04/30 | 1517 | 98.8 | 4 | 0.3 | 2 | 0.1 | 12 | 0.8 | 1535 | 2 |
| 2022/05/01 | 1559 | 99.6 | 2 | 0.1 | 0 | 0 | 4 | 0.3 | 1565 | 2 |
| 2022/05/20 | 1368 | 99.4 | 2 | 0.1 | 2 | 0.1 | 4 | 0.3 | 1376 | 8 |
| 2022/05/30 | 1269 | 99.8 | 1 | 0.1 | 1 | 0.1 | 1 | 0.1 | 1272 | 2 |
| 2022/10/31 | 1149 | 99.5 | 0 | 0 | 4 | 0.3 | 2 | 0.2 | 1155 | 6 |
| 2022/11/01 | 1602 | 98.6 | 0 | 0 | 16 | 1 | 7 | 0.4 | 1625 | 6 |
| 2022/11/02 | 1113 | 98.1 | 0 | 0 | 18 | 1.6 | 3 | 0.3 | 1134 | 6 |
| 2022/11/03 | 1509 | 98.9 | 1 | 0.1 | 16 | 1 | 0 | 0 | 1526 | 6 |
| 2022/11/04 | 1743 | 99.9 | 0 | 0 | 2 | 0.1 | 0 | 0 | 1745 | 6 |
| 2022/11/07 | 1410 | 99.7 | 0 | 0 | 4 | 0.3 | 0 | 0 | 1414 | 9 |
| 2022/11/08 | 2586 | 99.8 | 2 | 0.1 | 2 | 0.1 | 1 | 0 | 2591 | 9 |
| 2022/11/09 | 2369 | 99.9 | 0 | 0 | 3 | 0.1 | 0 | 0 | 2372 | 9 |
| 2022/11/10 | 1899 | 99.7 | 0 | 0 | 6 | 0.3 | 0 | 0 | 1905 | 6 |
| 2022/11/11 | 1266 | 99.9 | 0 | 0 | 0 | 0 | 1 | 0.1 | 1267 | 6 |
| 2022/11/12 | 1791 | 99.7 | 0 | 0 | 2 | 0.1 | 3 | 0.2 | 1796 | 9 |
| 2022/11/13 | 1830 | 100 | 0 | 0 | 0 | 0 | 0 | 0 | 1830 | 9 |
| 2022/11/14 | 1368 | 99.1 | 4 | 0.3 | 7 | 0.5 | 1 | 0.1 | 1380 | 3 |
| 2022/11/15 | 1275 | 98.8 | 1 | 0.1 | 12 | 0.9 | 2 | 0.2 | 1290 | 9 |
| 2022/11/16 | 1175 | 97.7 | 7 | 0.6 | 18 | 1.5 | 3 | 0.2 | 1203 | 9 |
| 2022/11/17 | 1120 | 96.1 | 6 | 0.5 | 37 | 3.2 | 2 | 0.2 | 1165 | 12 |
| 2022/11/25 | 1557 | 98.7 | 5 | 0.3 | 11 | 0.7 | 4 | 0.3 | 1577 | 4 |
| 2022/11/26 | 1131 | 99 | 9 | 0.8 | 3 | 0.3 | 0 | 0 | 1143 | 4 |
| 2022/12/18 | 1161 | 98.6 | 11 | 0.9 | 5 | 0.4 | 1 | 0.1 | 1178 | 4 |
| 2022/12/20 | 1119 | 92.5 | 39 | 3.2 | 31 | 2.6 | 21 | 1.7 | 1210 | 4 |
| 2023/01/31 | 1512 | 100 | 0 | 0 | 0 | 0 | 0 | 0 | 1512 | 3 |
| 2023/02/01 | 1321 | 100 | 0 | 0 | 0 | 0 | 0 | 0 | 1321 | 5 |
| 2023/02/02 | 2140 | 99.8 | 3 | 0.1 | 2 | 0.1 | 0 | 0 | 2145 | 5 |
| 2023/02/03 | 1309 | 99.6 | 4 | 0.3 | 1 | 0.1 | 0 | 0 | 1314 | 5 |
| 2023/02/04 | 1200 | 99.5 | 5 | 0.4 | 1 | 0.1 | 0 | 0 | 1206 | 1 |
| 2023/02/05 | 1100 | 99.1 | 7 | 0.6 | 3 | 0.3 | 0 | 0 | 1110 | 1 |
| 2023/02/06 | 1953 | 99.7 | 4 | 0.2 | 2 | 0.1 | 0 | 0 | 1959 | 1 |
| 2023/02/07 | 1698 | 99.6 | 5 | 0.3 | 1 | 0.1 | 0 | 0 | 1704 | 1 |
| 2023/02/08 | 1698 | 100 | 0 | 0 | 0 | 0 | 0 | 0 | 1698 | 1 |
| 2023/02/09 | 1995 | 100 | 0 | 0 | 0 | 0 | 0 | 0 | 1995 | 1 |
| 2023/02/11 | 1134 | 98.1 | 11 | 1 | 9 | 0.8 | 2 | 0.2 | 1156 | 1 |
| 2023/02/12 | 1209 | 97.7 | 8 | 0.6 | 17 | 1.4 | 4 | 0.3 | 1238 | 1 |

**Supplementary Table 2:** Results of the two-proportion z-test used to test whether the proportion of high-risk days in a Circulation Weather Type (CWT) group is larger than the overall proportion of high-risk days across other CWTs. For the p-values, an * indicates that the value is statistically significant at the 5% alpha level, while a ** indicates that the value is statistically significant at the 10% alpha level.

| CWT group | Z-statistic | p-value |
| --- | --- | --- |
| 1 | 0.97 | 0.046* |
| 2 | 4.26 | 0.980 |
| 3 | 0.92 | 0.831 |
| 4 | 3.28 | 0.035* |
| 5 | 0.86 | 0.077** |
| 6 | 0.02 | 0.446 |
| 7 | 1.98 | 0.920 |
| 8 | 0.20 | 0.328 |
| 9 | 0.02 | 0.438 |
| 10 | 0.32 | 0.287 |
| 11 | 1.16 | 0.859 |
| 12 | 2.13 | 0.928 |
| 13 | 0.13 | 0.357 |
| 14 | 0.75 | 0.193 |


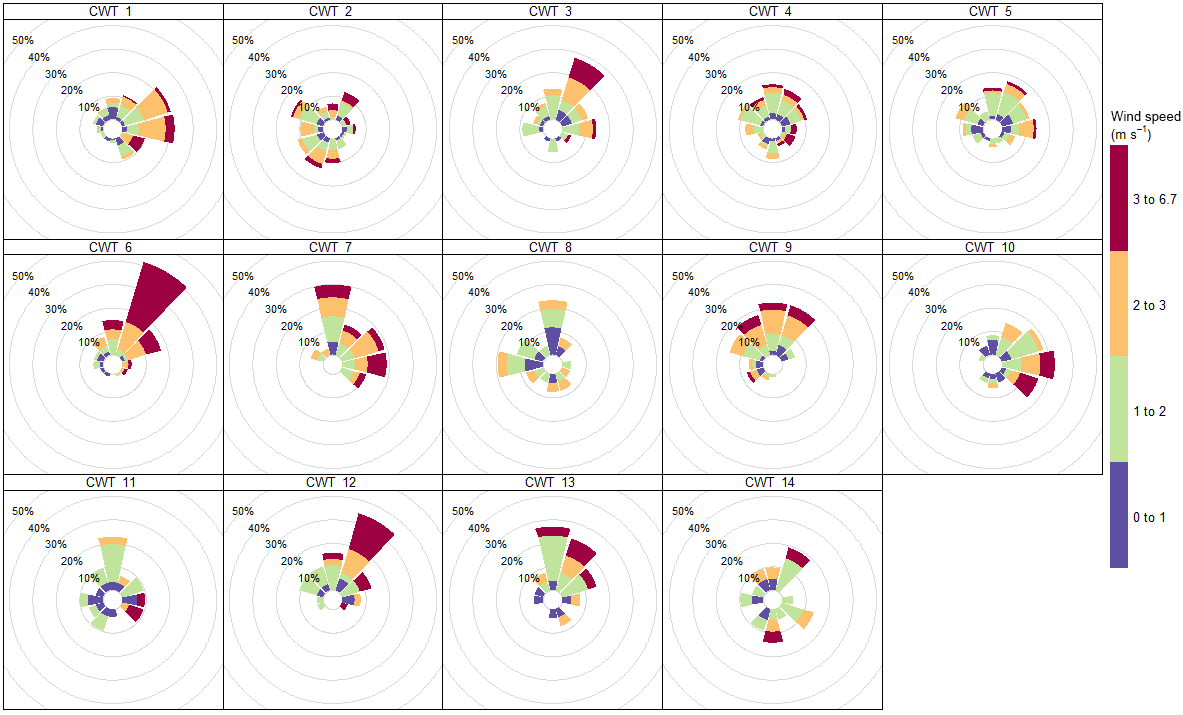
**Supplementary Figure 2:** Windrose plots per Circulation Weather Type (CWT).





**Supplementary Figure 3:** Boxplots depicting the annual cycle of the number of daily aerospora grains grouped by month for 08/2019-02/2023 – plot a, b, c and d are for aerospora types grouped as fungal spores, grass, trees, and weeds, respectively. Grey dots represent the mean number of daily aerospora grains per month, while the whiskers extend to the minimum and maximum number of daily aerospora grains.
